# Academic burnout among master and doctoral students during the COVID-19 pandemic

**DOI:** 10.1038/s41598-023-31852-w

**Published:** 2023-03-23

**Authors:** Diego Andrade, Icaro J. S. Ribeiro, Orsolya Máté

**Affiliations:** 1 Doctoral School of Health Sciences, Faculty of Health Sciences, University of Pécs, Pécs, Hungary; 2 Nursing Faculty, Southwest Bahia State University, Jequié, Brazil

**Keywords:** Psychology, Public health

## Abstract

The COVID-19 outbreak has had an immense impact on academic life and public health. Graduate students had experienced obligatory curfews and quarantines due to the COVID-19 outbreak directly impacting their mental health and triggering academic burnout. In this cross-sectional study, we address the issue of mental health in graduate students by relating it to the factors associated with burnout syndrome during the COVID-19 pandemic. A total of 519 graduate students from master's and Ph.D./DLA degrees across universities in Hungary and other European countries participated in this study. The Copenhagen burnout inventory student version was used to evaluate burnout syndrome as an outcome. Our findings displayed burnout significantly lower among graduate students who had good sleep quality, receive high levels of support from their university, and were satisfied with how their university dealt with the pandemic. The excessive consumption of alcohol, the use of antidepressants, being single, and thinking about dropping out showed as predictive factors of burnout. The results add to emergent evidence on the impact of the COVID-19 pandemic on mental health and the predicted factors of academic burnout among master and doctoral students.

## Introduction

The COVID-19 pandemic has forced universities worldwide to adopt strong measures through compulsory confinement and social isolation. As a result, a negative impact on many students’ mental health was observed, such as the development of academic burnout^1–3^.

Studies has shown that graduate students are more affected by mental health issues and more likely to present higher levels of stress than undergraduate students and the general population during COVID-19^4,5^. With the COVID-19 pandemic, new demands have arisen that directly interfere on students' mental health, such as interruption of learning, uncertainty about prolongation of research duration, laboratory closures, losing part-time teaching job, expiring visas for foreign students, uncertain of funding/grant discontinuation, inadequate mentoring, lack of concentration at home and performing more household chores^1,6,7^. These academic disruptions combined with significant apprehension about the completion and quality of the work, career concerns, usual high workload even at home, colleagues’ competition, inadequate university support, insufficient supervision, financial issues, low autonomy, emotional suffering, academic dissatisfaction, are examples of factors that predispose to a chronic stress and result in academic burnout^8–16^.

Burnout is described as a psychological disorder emerged as a response to chronic emotional and interpersonal stressors in the working environment, composed of emotional exhaustion, depersonalization and reduced personal accomplishment^17^. Emotional exhaustion refers to depletion of emotional resources by feeling emotionally overextended, exhausted and drained. Depersonalization is often referring to cynicism, and includes negative attitudes toward other people, like colleagues, patients, or clients. A reduction in personal accomplishment refers to decreased satisfaction and declined feeling of competence and successful achievement^18^. The development of burnout in students is directly related to excessive and prolonged stress caused by piling school work and academic demands combined with drained energy, reduced enthusiasm toward academic tasks, lack of positive attitudes and low academic achievements^19,20^.

In addition to all of the academic pressure, graduate students also have had to deal with the COVID-19 outbreak, experiencing curfews, social isolation, and quarantines. In these stressful circumstances, studying and conducting research can have a wide range of effects on mental health, which can lead to academic burnout^16,21–23^.

The study of this issue may be of special international interest, considering the recent COVID-19 pandemic, that by itself addresses a critical and unique gap in research concerning the mental status in regard to academic burnout among graduate students in Hungary and some European countries. We aim to address the issue of mental health in graduate students by relating it to the factors associated with burnout syndrome during the COVID-19 pandemic.

## Results

A total of 519 students (365 women [70.30%]) with a mean age of 31 years (± 7.76) were included in the evaluation. Single individuals (55.3%), from non-European countries (43.4%), from the University of Pécs (49.1%) and with PhD/DLA educational level (56.3%) prevailed (Table 1).

**Table 1:** Sociodemographic characteristics of 519 evaluated graduate students.

	N	%
Sex		
Male	154	29.7
Female	365	70.3
Marital status		
Single	287	55.3
Married	123	23.7
Other	109	21.0
Origin country		
Hungary	183	35.3
European countries	111	21.4
Non European countries	225	43.4
University		
Univesity of Pécs	255	49.1
University of Szeged	44	8.5
ELTE	50	9.6
University of Debrecen	35	6.7
University of Miskolc	30	5.8
Others	105	20.2
Currently level of education		
PhD/DLA degree	292	56.3
Master’s degree	227	43.7

Higher averages of burnout were observed in all the dimensions of the CBI for females, with a significant difference for the dimensions CRB (CRB, *p* ≤ 0.01). With regard to marital status, all dimensions of the CBI were higher among singles and the difference was significant for all dimensions (PB, *p* = 0.04; SRB, *p* ≤ 0.01; CRB, *p* = 0.02; TRB, *p* ≤ 0.01) (Table 2). Evaluating aspects related to academic life, those who thought often about dropping out of the course had higher levels of burnout for all dimensions. With regard to how the university dealt with the pandemic, burnout was more frequent among those dissatisfied with the strategies used. Finally, among those who did not feel supported by the university, burnout was also higher (Table 3).

**Table 2:** Comparison of the mean of the CBI dimensions according to sociodemographic characteristics.

	PB mean (SD)	*p*	SRB mean (SD)	*p*	CRB mean (SD)	*p*	TRB mean (SD)	*p*
Sex^a^		0.09		0.36		< 0.01		0.36
Male	54.05 (16.76)		49.69 (19.41)		37.20 (22.17)		34.31 (26.14)	
Female	57.11 (19.75)		51.57 (21.09)		30.67 (21.45)		32.10 (24.74)	
Marital status^b^		0.04		< 0.01		0.02		< 0.01
Single	57.97 (18.99)		53.29 (20.34)		34.32 (21.03)		35.34 (25.49)	
Married	52.81 (18.24)		44.16 (20.89)		27.84 (22.29)		26.22 (23.99)	
Other	55.39 (19.21)		52.56 (19.47)		33.48 (22.88)		33.33 (24.47)	

^a^t-test; ^b^ANOVA.

**Table 3:** Comparison of the average of the CBI dimensions according to academic characteristics.

	PB mean (SD)	*p*	SRB mean (SD)	*p*	CRB mean (SD)	*p*	TRB mean (SD)	*p*
How often do you think about quitting your course?^b^		< 0.01		< 0.01		< 0.01		< 0.01
Frequently	65.77 (20.48)		60.71 (21.32)		42.11 (23.97)		47.63 (27.45)	
Sometimes	58.72 (16.35)		56.77 (17.80)		37.70 (21.32)		35.23 (22.93)	
Never	52.23 (19.15)		44.77 (20.22)		26.93 (20.04)		27.54 (24.42)	
I am satisfied how my university is dealing with the pandemic^b^		0.13		0.01		< 0.01		< 0.01
Strongly disagree	63.33 (22.96)		61.78 (24.55)		51.04 (25.79)		48.33 (28.97)	
Disagree	57.73 (16.53)		53.61 (19.19)		37.31 (20.42)		39.26 (25.00)	
Neutral	54.88 (20.91)		51.56 (19.78)		34.40 (21.69)		37.89 (27.08)	
Agree	57.37 (17.13)		51.35 (20.38)		33.79 (20.91)		32.97 (22.97)	
Strongly agree	53.56 (20.25)		46.51 (21.15)		23.49 (20.23)		21.41 (21.68)	
I feel supported by my university during the COVID pandemic^b^		< 0.01		< 0.01		< 0.01		< 0.01
Strongly disagree	65.13 (18.33)		61.10 (21.60)		40.76 (24.77)		47.28 (28.18)	
Disagree	52.82 (15.78)		50.29 (16.99)		39.89 (19.88)		40.04 (23.88)	
Neutral	58.06 (19.30)		54.66 (22.11)		33.02 (20.91)		32.97 (24.29)	
Agree	55.09 (18.51)		49.07 (19.60)		32.74 (22.25)		31.99 (23.49)	
Strongly agree	55.11 (20.64)		46.91 (21.14)		23.59 (19.13)		22.65 (23.72)	

^b^ANOVA.

Excessive alcohol consumption (CRB, *p* ≤ 0.01) and use of antidepressants (CRB, *p* ≤ 0.01; TRB, *p* = 0.04) were also associated with higher levels of burnout, but only for the CRB and TRB dimensions. With regard to sleep quality, among those who rated it as poor, they had higher levels of burnout for the PB and SRB dimensions (PB, SRB, *p* ≤ 0.01) (Table 4).

**Table 4:** Comparison of the mean of the CBI dimensions according to health and life habits characteristics.

	PB mean (SD)	*p*	SRB mean (SD)	*p*	CRB mean (SD)	*p*	TRB mean (SD)	*p*
Alcohol consumption^b^		0.89		0.61		< 0.01		0.11
Excessively	56.25 (12.03)		41.07 (2.06)		62.50 (14.43)		56.25 (2.40)	
Moderately	56.49 (18.95)		51.19 (20.22)		33.56 (22.19)		31.83 (25.03)	
No consumption	55.65 (19.15)		50.78 (21.58)		30.05 (20.71)		34.03 (25.45)	
Antidepressant medications^a^		0.36		0.17		< 0.01		0.04
Yes	59.07 (18.89)		55.67 (21.36)		42.89 (23.38)		41.29 (29.91)	
No	56.00 (18.96)		50.65 (20.54)		31.89 (21.58)		32.16 (24.72)	
Sleep quality^b^		< 0.01		< 0.01		0.37		0.44
Poor	66.78 (22.21)		60.42 (23.63)		38.08 (24.98)		33.68 (25.24)	
Regular	59.44 (17.55)		53.51 (19.37)		33.33 (21.69)		35.07 (24.73)	
Good	53.75 (18.03)		49.09 (20.75)		31.67 (21.71)		31.02 (24.25)	
Very good	51.06 (20.62)		46.21 (19.49)		31.22 (20.95)		32.59 (29.24)	

## Discussion

The current study discloses the influence that the COVID-19 pandemic has had on the mental health of graduate students by analysing the factors associated with burnout syndrome. We analysed sociodemographic, academic, health and life habits factors. We found that being single had an effect in all burnout domains and the sex female as well with the colleagues related burnout domain along the sociodemographic characteristics. Among the academic characteristics, we found high levels of academic burnout for all dimensions among those who had university drop-out intentions, were dissatisfied with how the university dealt with the pandemic and also those who did not feel supported by the university during the outbreak. We found high levels of academic burnout among colleagues-related burnout and teacher-related burnout dimensions in the health and life habits characteristics with those who had excessive alcohol consumption and took antidepressants. The personal burnout and studies-related burnout presented a high level among those who had a bad sleep quality.

Sex differences associated with burnout is still without a literature consensus. Some authors suggest that females are more likely suffer from exhaustion and have higher levels of stress than males, while others report no difference between the two sexes regarding exhaustion and stress^9,24^. Nonetheless, our study found association between sex and the colleagues-related burnout dimension, corroborating that females are more likely to develop burnout. Studies has been reporting^25–27^ that the higher chronic stress level among females is influenced not only by the university environment that includes role conflict, excessive workload, competitive colleagues and considerable mental pressure to publish, but also by inappropriate behaviours, such as harassment, bullying and gender discrimination.

Woolston^28^ published a study by the Nature’s survey with 6.296 PhD respondents, one-quarter of who identified as female reported personally experiencing harassment or discrimination compared with 16% of those identifying as men. Moreover, 57% of students who experienced bullying reported fear of personal repercussions if they discuss their situation. This discloses that sex differences associated with burnout exist and being a woman researcher is still a challenge.

We also found that being single was associated with higher burnout scores in all dimensions compared to those with married or other marital status. This result was well reported by Maslach et al.^17^ that found higher burnout among those who identified as single rather than married. Among postgraduates, other study also found higher burnout scores in single individuals compared to the married ones^14^. Marriage as a social support may act as a protecting factor from chronic stress and can play a role in reducing academic burnout.

Evaluating aspects related to academic life, we found that many graduate students at some point had considered abandoning their studies. Several studies have reported high rates of university withdrawal intentions, for example, 30–70% of doctorate students will may not complete their PhD degree^8,29–31^. Experiences of high stress, anxiety and exhaustion, demonstrated a lack of interest in their studies which appears to influence drop-out intentions^8,32,33^. In contrast, satisfaction and engagement in research, supervision from several supervisors, integration and networks in the research community has a reverse effect in reducing burnout rates and enhancing success to degree completion^34,35^. This reveals that the decision to drop out of studies has a direct influence on burnout experiences, as shown by an association in all four burnout’s dimensions.

Furthermore, relating to the academic life, we found that the feeling of not being supported by the university during the COVID-19 outbreak had an association with all burnout dimensions. The academic support that graduate students receive from their department, faculty or university is essential to develop the sense of belonging and fitting in the educational environment. The lack of this perceived organizational support can increase the risk of experiencing exhaustion and the dissatisfaction with the doctoral studies, leading to academic burnout and consequences such as the intention to leave the degree^8,36,37^. The dissatisfaction with how the university dealt with the pandemic was another result found associated with the dimensions studies-related burnout, colleague-related burnout and teacher-related burnout, showing the direct influence of the institution, work environment and supervision on student satisfaction and well-being^37–39^. Particularly, the perceived organizational support and satisfaction with the institution can be decreased when the graduate students need to deal with the lack of transparency, undefined career prospects, unclear expectations during an outbreak such the COVID-19, thus the aforementioned factors can raise the risk for developing burnout.

Analysing the health and life habits characteristics we found that a bad sleep quality is associated with two burnout dimensions, personal burnout and studies-related burnout. Allen et al. also found in their study with graduate students that sleep quality has more consistent relationship with burnout and might be more important than sleep duration in order to reduce burnout levels^40^. It is already known that a poor sleep quality is associated with higher levels of fatigue and exhaustion, and when it comes to graduate students, this can impact directly and negatively the student’s personal life and academic productivity^41,42^. Given that together with prolonged and chronic stress, the lack of energy and motivation can make students less interested in their studies and more prone to develop academic burnout.

Moreover, with regards to the health and life habits factors related to academic burnout, we found that self-reported excessive alcohol consumption and use of antidepressants are both associated with colleague-related burnout and teacher-related burnout. The association between burnout syndrome and the consumption of alcohol has been widely reported, although a limited number of studies have examined this relationship among graduate students. The vulnerable situations of the students, emotional conflicts in the academia environment, excess of activities and competitiveness are pointed as the most contributing factors in the development of high levels of stress and alcohol misuse. This excessive alcohol consumption may be viewed as a dysfunctional coping mechanism, since the students may abuse alcohol as a strategy for regulating tension and stressful situations in the academia^43–46^.

The use of antidepressants can be also observed as a way of coping with adversity in the academia. It is already known that the academic stressors are related to stress, anxiety, depression, and when combined with extra load on studying as well as the need to enhance performance and concentration, students may resort to the use of antidepressants to avoid episodes of social anxiety and depressive behaviour. This finding is consistent with other studies, that reported students who use antidepressants, present high levels of burnout^47–49^. The misuse of alcohol and/or other substances are linked with burnout, and by neglecting that, it can lead to serious consequences.

### 1Limitations

Our study has some important limitations. The cross-sectional study design limited our ability to establish causality between the associations. The online assessment to collect data during the COVID-19 outbreak may carry response bias and are less reliable. Therefore, we have used screening tools in this study and our findings should be interpreted carefully, since it is not a clinical psychiatric diagnostic instrument.

## Conclusion and implications

This study analysed a number of factors thought to influence graduate students to develop academic burnout during the COVID-19 pandemic. Burnout showed significantly lower among graduate students who receive high levels of support from their university, were satisfied with how their university dealt with the pandemic and had a good sleep quality. The excessive consumption of alcohol, the use of antidepressants, being single and thinking about abandoning the university had a negative impact academic success and were predictory to burnout. We believe that these findings can offer patterns and predictors for future graduate students and university administrators to identify, promote and implement changes to help those who are facing the academic burnout and prevent other graduate students from develop it.

## Methods

### Study design and data collection

This current study is a cross-sectional analytical research. Data were collected through an online survey between September 2021 and March 2022. We tested a pilot of our preliminary instrument to ensure question clarity, and confirm completion of the survey in approximately 15 min. Data collection was done by virtual distribution over the Google Forms platform, along a close co-operation with international associations of graduate students and university departments. The form was disseminated through emails and included an invitation to participate, social media channels from communities for graduate students, and by asking participants to pass along the survey link to other eligible participants. The survey was designed and carried out in accordance with the Checklist for Reporting Results of Internet E-Surveys (CHERRIES)^50^ (Supplementary Table S1).

Participation was anonymous and voluntary throughout the entire study period, and they were informed about the research and goal before giving their consent. We were unable to assess how many people viewed the online invitation, and therefore we could not determine the response rate of the study. Altogether, 542 students participated in the study. After eliminating incomplete answers, the final sample consisted of 519 graduate students which yielded a 95.75% completion rate.

Inclusion criteria were graduate students at master or Ph.D./DLA level by voluntary participation. Exclusion criteria were incomplete questionnaires and those who did not wish to participate in the research. The pilot test data and incomplete questionnaires with missing responses were excluded from the study.

### Measures

The dependent variable was academic burnout syndrome, which was evaluated through the Copenhagen burnout inventory—student version^51^. The CBI was developed by Kristensen et al.^51^, and adapted for students by Campos et al.^52^. This scale consists of 25 items that represent 4 subscales: Personal Burnout (PB), Studies-related Burnout (SRB), Colleague-related Burnout (CRB), and Teacher-related Burnout (TRB). The answers are quantifying as 100, 75, 50, 25, and 0% respectively, with a reverse scoring for item 10. We used the Kristensen’s criteria of burnout score, 50–74 is consider moderate, 75–99 is high, and a score of 100 consider as severe burnout^53^. In the current study, the Cronbach’s alpha for the CBI-S scale was 0.93, indicating good internal reliability.

All the other selected variables were classified according to sociodemographic, academic and health status by self-reported answers.

- Sociodemographic variables: age, sex (male, female, prefer not to mention), marital status (single, married, other), education level (PhD/DLA, master’s), and origin country (Hungary, European, non-European).
- Academic variables: university of origin, study year, university’s drop-out intention (3-point scale ranging from: frequently; sometimes; never), university’s satisfaction during COVID-19 pandemic (5-point scale ranging from: strongly disagreed; disagree; neutral; agreed; strongly agreed), university’s support during COVID-19 pandemic (5-point scale ranging from: strongly disagreed; disagree; neutral; agreed; strongly agreed).
- Health status and life habits variables: alcohol consumption (excessively; moderately; no consumption), antidepressant medications in use (yes; no), and quality of sleep (4-point scale ranging from: poor; regular; good; very good).

### Statistical analysis

Statistical analysis was performed using initially the Microsoft Excel for Microsoft 365 (Microsoft Corp., Redmond, WA, USA). A *p* value of 0.05 (two-tailed) was considered to be statistically significant. Descriptive statistics were performed with the calculation of the mean (M) and standard deviation (SD) for quantitative variables, and percentages were calculated for qualitative variables.

In order to verify the difference between the means of the CBI dimensions and the independent variables, the t test (two groups) and the ANOVA (more than two groups) were applied, given the normality of the data attested by the Kolmogorov Smirnov test. All analyses were performed using the Stata statistical package version 12 (Stata Corp., College Station, TX, USA), with a significance level of 5%.

### Ethical considerations

The study was approved Ethical Committee from the University of Pécs approved the study, under protocol number 8471, and also respected the Helsinki guidelines at all times. All participants statement an informed consent before becoming part of this study.

## Supplementary Information

Supplementary Table S1.

## Data Availability

The datasets used and/or analysed during the current study available from the corresponding author on reasonable request.
