# Supplementary material for: Academic burnout among master and doctoral students during the COVID-19 pandemic
Source: Sci Rep. 2023 Mar 23;13:4745. doi: 10.1038/s41598-023-31852-w (PMC10034888; doi:10.1038/s41598-023-31852-w)
Supplement: Supplementary file 1 — Supplementary Table S1. [file 41598_2023_31852_MOESM1_ESM.docx]

**Supplementary Table S1**: CHERRIES-Checklist for reporting results of Internet-E-Surveys.

Reference: Eysenbach G. (2004) Improving the quality of Web surveys: the Checklist for Reporting Results of Internet E-Surveys (CHERRIES) *J Med Internet Res* **6**(3):e34 doi:10.2196/jmir.6.3.e34

| Item Category | Checklist Item | Explanation |
| --- | --- | --- |
| Design | Describe survey design | The target population was masters and Ph.D./DLA students. The study involved a convenience sample. Inclusion criteria were graduate students at the master or Ph.D./DLA level by voluntary participation. The exclusion criteria were incomplete questionnaires. |
| IRB | IRB approval | Yes. The study protocol was approved by the ethics committee of University of Pécs. |
|  | Informed consent | On the welcome page of the online survey, participants were informed about the research and the informed consent was obtained from all those agreeing to answer the survey. It was display that the survey would take approximately 15 minutes to complete, that all responses were confidential and anonymous. Consent was indicated when respondents clicked the ‘I consent’ button at the bottom of this same page. |
|  | Data protection | No personal information was collected (Random IDs were given to participants in case they wished to withdraw their data). |
| Development and pretesting | Development and testing | The survey was designed using input from earlier research. The survey was pre-tested by a pilot study, adapted and approved by the research team. |
| Recruitment process and description of the sample having access  to the questionnaire | Open survey versus closed survey | Open survey |
|  | Contact mode | The form was disseminated through emails and social media channels from communities for graduate students including an invitation to participate. We also asked consented participants to pass along the survey link to other eligible participants. |
|  | Advertising the  survey | Email invitation was sent to several coordinators of various graduate groups and organizations with the link of the survey to be disseminate to their listservs. Also, a message was posted on the social media channels such Facebook and Instagram of graduate student’s pages inviting eligible participants to complete the survey |
| Survey administration | Web/E-mail | Web-based survey, hosted by the Google Forms platform. Data was entered automatically when participants responded to the questions. |
|  | Context | Google Form platform. |
|  | Mandatory/  Voluntary | Voluntary |

|  | Incentives | None |
| --- | --- | --- |
|  | Time/Date | September 2021 to March 2022 |
|  | Randomization of items or  questionnaires | No randomization of items was used. |
|  | Adaptive questioning | Adaptive questioning was used. |
|  | Number of items | 2 to 9 |
|  | Number of screens  (pages) | 6 screens |
|  | Completeness check | In case of incomplete answers, there was an alert before leaving the survey page on which the item was contained. Most items, except screener items and those items required for adaptive questioning included a “I don’t know” or “I prefer not to say” option. |
|  | Review step | Participants were able to change their responses by using a “Back” button at the bottom of each screen. |
| Response rates | Unique site visitor | Only participants or visitors completing at least the first page and proceeding to the next page were counted. Thus, calculation of views or participation  rates were not possible. |
|  | Completion rate | Of the 542 respondents who started the survey, 519 completed it, giving a completion rate of 95.75%. |
| Preventing multiple entries from the same  individual | Cookies used | Cookies were not used. |
|  | IP check | Not used to maintain anonymity. |
|  | Log file analysis | The study did not include a log file analysis. |
|  | Registration | No registration required. |
| Analysis | Handling of incomplete questionnaires | Only completed questionnaires were included in the analysis. |
|  | Questionnaires submitted with an  atypical timestamp | Average time of access was recorded, but no respondents were removed from the survey for a minimal time answer. |
|  | Statistical correction | No statistical correction was performed. |
